# Perturbed Hippocampal Synaptic Inhibition and γ-Oscillations in a Neuroligin-4 Knockout Mouse Model of Autism

**DOI:** 10.1016/j.celrep.2015.09.011

**Published:** 2015-10-08

**Authors:** Matthieu Hammer, Dilja Krueger-Burg, Liam Patrick Tuffy, Benjamin Hillman Cooper, Holger Taschenberger, Sarit Pati Goswami, Hannelore Ehrenreich, Peter Jonas, Frederique Varoqueaux, Jeong-Seop Rhee, Nils Brose

**Affiliations:** 1 Department of Molecular Neurobiology, Max Planck Institute of Experimental Medicine, Hermann-Rein-Straße 3, 37075 Göttingen, Germany; 2 Clinical Neuroscience, Max Planck Institute of Experimental Medicine, Hermann-Rein-Straße 3, 37075 Göttingen, Germany; 3 Institute of Science and Technology Austria, Am Campus 1, 3400 Klosterneuburg, Austria

## Abstract

Loss-of-function mutations in the synaptic adhesion protein Neuroligin-4 are among the most common genetic abnormalities associated with autism spectrum disorders, but little is known about the function of Neuroligin-4 and the consequences of its loss. We assessed synaptic and network characteristics in Neuroligin-4 knockout mice, focusing on the hippocampus as a model brain region with a critical role in cognition and memory, and found that Neuroligin-4 deletion causes subtle defects of the protein composition and function of GABAergic synapses in the hippocampal CA3 region. Interestingly, these subtle synaptic changes are accompanied by pronounced perturbations of γ-oscillatory network activity, which has been implicated in cognitive function and is altered in multiple psychiatric and neurodevelopmental disorders. Our data provide important insights into the mechanisms by which Neuroligin-4-dependent GABAergic synapses may contribute to autism phenotypes and indicate new strategies for therapeutic approaches.

## Introduction

Autism spectrum disorders (ASDs) are among the most prevalent neuropsychiatric disorders, with a high heritability that indicates a major role of genetic causes (Chen et al., 2015). Indeed, numerous ASD-associated genetic abnormalities, including monogenic heritable forms of ASD, have been identified, and many of these affect proteins that determine the structure and function of neuronal synapses, which led to the notion that ASD may be a synaptopathy (Kleijer et al., 2014; Zoghbi, 2003). Among the most common monogenic causes associated with ASD are mutations in the *NLGN4* gene (Jamain et al., 2003; Laumonnier et al., 2004), which encodes a member of the Neuroligin (Nlgn) family of synaptic cell adhesion proteins (Krueger et al., 2012). More than 50 mutations in *NLGN4* have been identified in individuals with ASD (Chanda et al., 2015; Kleijer et al., 2014; Südhof, 2008), most of which lead to a loss of function (Zhang et al., 2009). Corroborating this, Nlgn4 knockout (KO) mice show behavioral changes reminiscent of the core symptoms of ASD, including impairments in social interactions and communication as well as repetitive behaviors and interests (El-Kordi et al., 2013; Jamain et al., 2008; Ju et al., 2014).

Rodent Nlgn4 is expressed throughout the CNS and localizes specifically to inhibitory synapses in retina, brainstem, and spinal cord (Hoon et al., 2011). Like its homolog Nlgn2, Nlgn4 can activate the signaling protein Collybistin (CB) and thereby recruit the scaffold protein Gephyrin (GPH) and inhibitory neurotransmitter receptors to nascent synapses (Hoon et al., 2011). Correspondingly, Nlgn4 deletion in mice causes aberrant inhibitory neurotransmission at retinal synapses (Hoon et al., 2011). While these findings are consistent with the notion that alterations in the balance of excitatory and inhibitory synaptic transmission play a role in ASD (Rubenstein and Merzenich, 2003; Yizhar et al., 2011), only minimal information is available on the function of Nlgn4 in forebrain regions relevant to ASD symptoms and phenotypes. We addressed this issue by assessing synaptic and network characteristics in Nlgn4 KO mice, focusing on the hippocampus as a model brain region with a key role in cognition and memory. Our data demonstrate that Nlgn4 co-determines the structure and function of perisomatic inhibitory synapses in hippocampal area CA3, and that the subtle synaptic consequences of Nlgn4 loss can have dramatic consequences on network functions related to cognition.

## Results

### Nlgn4 Expression in the Mouse Hippocampus

**Figure 1:**
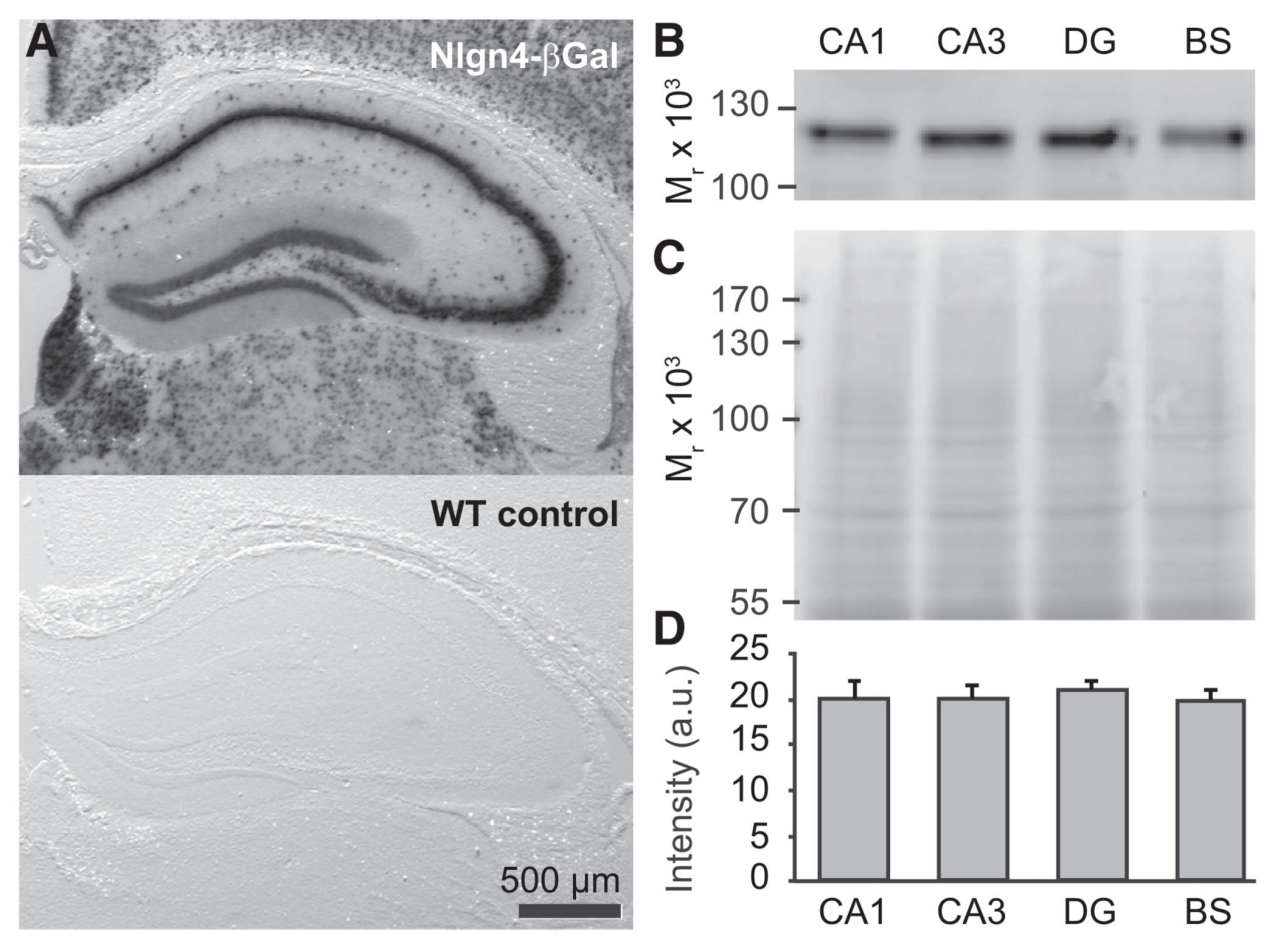
Nlgn4 Expression in the Hippocampus (A) (Top) Assessment of *Nlgn4* promoter activity using the β-galactosidase reporter expressed by the gene trap in the Nlgn4 KO line (Jamain et al., 2008) and X-gal staining reveal expression of the reporter throughout the hippocampus. (Bottom) WT control shows a lack of X-gal staining in the absence of β-galactosidase. (B–D) Nlgn4 levels in WT hippocampal CA1 and CA3 regions, dentate gyrus (DG), and brainstem (BS). Representative Nlgn4 immunoblot (B), corresponding protein loading (C, Memcode stain), and quantification (D) are shown (n = 5 animals).

As prior investigations of Nlgn4 expression were restricted to retina and brainstem (Hoon et al., 2011), we first characterized the expression of Nlgn4 in the mouse hippocampus, taking advantage of a gene trap in the Nlgn4 KO that leads to the expression of β-galactosidase under the control of the *Nlgn4* promoter (Jamain et al., 2008). β-galactosidase activity was observed in all discernible cell types throughout the juvenile (3 weeks old; Figure S1A) and adult hippocampus (8–12 weeks old; Figure 1A), and immunoblotting of tissue dissected from areas CA1, CA3, and the dentate gyrus (DG) showed that Nlgn4 protein is expressed at equal levels in all subregions of the adult hippocampus and at levels comparable to those in brainstem (Figures 1B–1D). While Nlgn4 in retina, brainstem, and spinal cord is specifically detectable at inhibitory synapses by immunostaining (Hoon et al., 2011), we failed to clearly detect Nlgn4 expression in the hippocampus and other forebrain areas by immunostaining, despite testing multiple antibodies and a wide array of fixation, antigen-retrieval, and staining protocols. In all cases, the Nlgn4 immunolabeling intensity in forebrain was much lower than in brainstem despite equal protein levels (Figures 1B and 1D), possibly due to differential posttranslational modifications of the Nlgn4 protein. Accordingly, we instead sought to determine the synapse specificity of Nlgn4 function by investigating the consequences of its deletion.

### Nlgn Isoform Expression in Nlgn4 KO Hippocampus

We first assessed the effect of Nlgn4 KO on synapse numbers and composition in the hippocampus. In immunoblot analyses of synaptosomes (Table S1) prepared from whole hippocampi of wild-type (WT) and Nlgn4 KO mice, we detected no significant differences in the levels of key components of inhibitory or excitatory synapses, indicating that any changes that might occur upon Nlgn4 loss must be too local or layer specific to be detectable in tissue homogenates that include all hippocampal regions. Interestingly, we observed a significant increase in the Nlgn2/Nlgn1 ratio in synaptosomes from Nlgn4 KOs, resulting from a modest increase in Nlgn2 levels and a decrease in Nlgn1 levels (Table S1). These changes indicate a possible compensatory role of other Nlgn isoforms in the absence of Nlgn4, most notably of Nlgn2, which has the same biochemical characteristics as Nlgn4 (Hoon et al., 2011). A corresponding upregulation of Nlgn4 upon loss of Nlgn2 was detected previously in inhibitory retinal synapses (Hoon et al., 2011).

### Inhibitory Synapses in the CA3 Stratum Pyramidale of Nlgn4 KO Hippocampus

**Figure 2:**
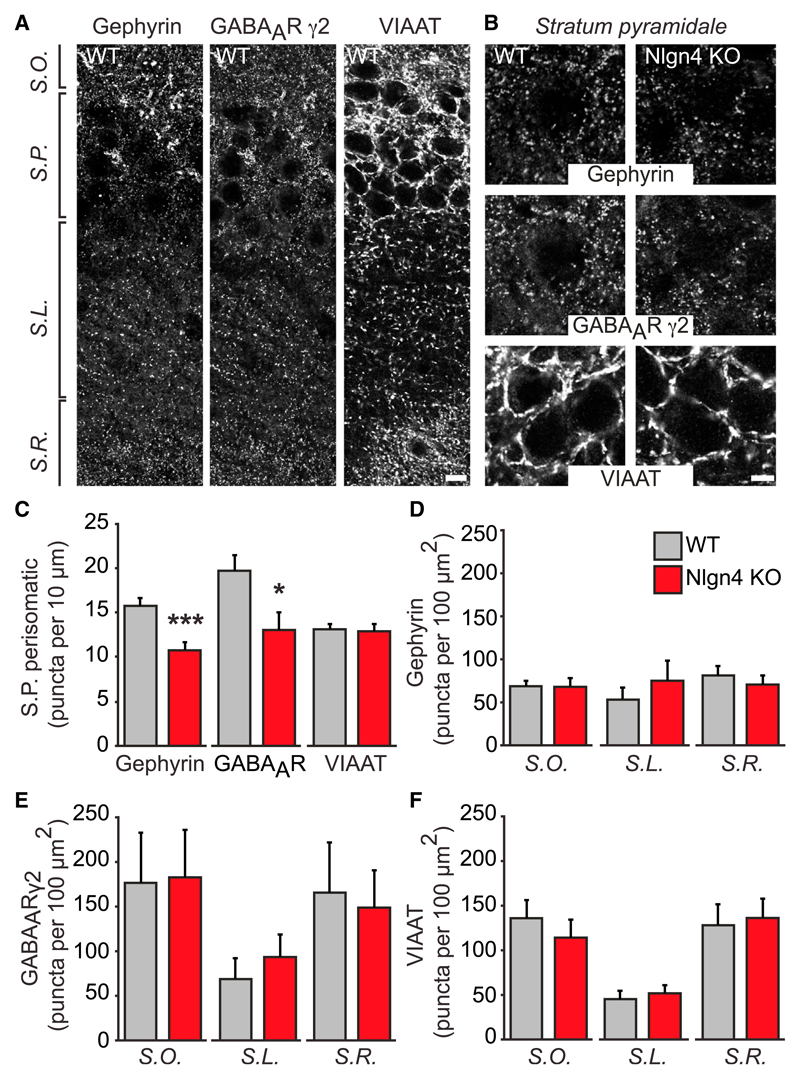
Inhibitory Synapses in the CA3 Stratum Pyramidale of Nlgn4 KOs (A) Images show GPH, GABA_A_Rγ2, and VIAAT immunoreactivity in CA3 of WT mice. Scale bar, 10 μm. (B) High-magnification images show GPH, GABA_A_Rγ2, and VIAAT immunoreactivity in CA3 stratum pyramidale of WT and Nlgn4 KO mice. Scale bar, 5 μm. (C) Quantification of GPH, GABA_A_Rγ2, and VIAAT puncta in the perisomatic area of pyramidal cells in CA3 is shown (puncta per 10 μm perimeter). (D–F) Quantifications of GPH (D), GABA_A_R γ2 (E), and VIAAT (F) puncta in the stratum oriens, stratum lucidum, and stratum radiatum of CA3 are shown (puncta per 100 μm^2^). S.O., stratum oriens; S.P., stratum pyramidale; S.L., stratum lucidum; S.R., stratum radiatum. Error bars represent SEM; n = 12 pairs of mice; *p < 0.05, ***p < 0.001.

To determine whether Nlgn4 KO causes local or layer-specific changes of synaptic protein levels in the hippocampus, we employed an immunostaining approach. It was shown previously that Nlgn4 KO leads to alterations in components of inhibitory postsynapses in the retina (Hoon et al., 2011). We thus performed immunostaining for GPH, a scaffold protein of inhibitory postsynapses, and for the GABA_A_ receptor γ2 subunit (GABA_A_Rγ2), a marker of mature inhibitory synapses, in the hippocampus of adult mice (Figures 2A and 2B). The density of GPH- and GABA_A_Rγ2-containing puncta in the CA3 region was decreased by 32% and 34%, respectively, in the perisomatic region of stratum pyramidale neurons of Nlgn4 KOs (Figures 2B and 2C). GPH puncta per 10 μm cell perimeter were as follows: WT, 15.8 ± 0.84; KO, 10.8 ± 0.88; n = 12, p < 0.001; and GABA_A_Rγ2 puncta per 10 μm cell perimeter were as follows: WT, 19.7 ± 1.72; KO, 13.1 ± 1.98; n = 12, p < 0.05. No alterations in the density of GPH or GABA_A_Rγ2 puncta were observed in other CA3 layers (Figures 2D and 2E). Similar reductions in GPH and GABA_A_Rγ2 puncta in CA3 stratum pyramidale were observed at a juvenile time point (3 weeks old; Figures S1B–S1D), indicating that the postsynaptic changes at inhibitory synapses caused by Nlgn4 KO arise early and are maintained after initial synapse formation.

To assess whether these changes are due to a decrease in the number of inhibitory synapses, we stained for the vesicular inhibitory amino acid transporter (VIAAT), a marker of inhibitory presynapses. No alterations in the number of VIAAT puncta were observed in the perisomatic region of the CA3 stratum pyramidale of Nlgn4 KOs (Figures 2B and 2C) or in any other layer (Figure 2F), indicating that Nlgn4 KO does not cause an overall loss of presynaptic innervation or decrease in the number of inhibitory synapses. Instead, it causes a defect in the synaptic recruitment of GPH and GABA_A_Rs, which also is seen upon KO of the Nlgn4-related Nlgn2 isoform (Poulopoulos et al., 2009; Soykan et al., 2014).

### Excitatory Synapses in the CA3 Stratum Pyramidale of Nlgn4 KO Hippocampus

To test whether Nlgn4 KO also affects excitatory synapses in the hippocampus, we stained for the excitatory synapse marker PSD-95. No alterations in the very sparse PSD-95 puncta density in the CA3 stratum pyramidale were observed, confirming that the changes in this layer are specific to inhibitory synapses (Figure S2). Similarly, no changes were observed in CA3 stratum oriens or stratum radiatum (Figure S2), while a small but significant decrease in the number of PSD-95 puncta was found in stratum lucidum (Figure S2B). These findings show that Nlgn4 KO may have differential effects even in different layers of the same brain region.

### Inhibitory Synaptic Transmission in the CA3 Region of Nlgn4 KO Hippocampus

**Figure 3:**
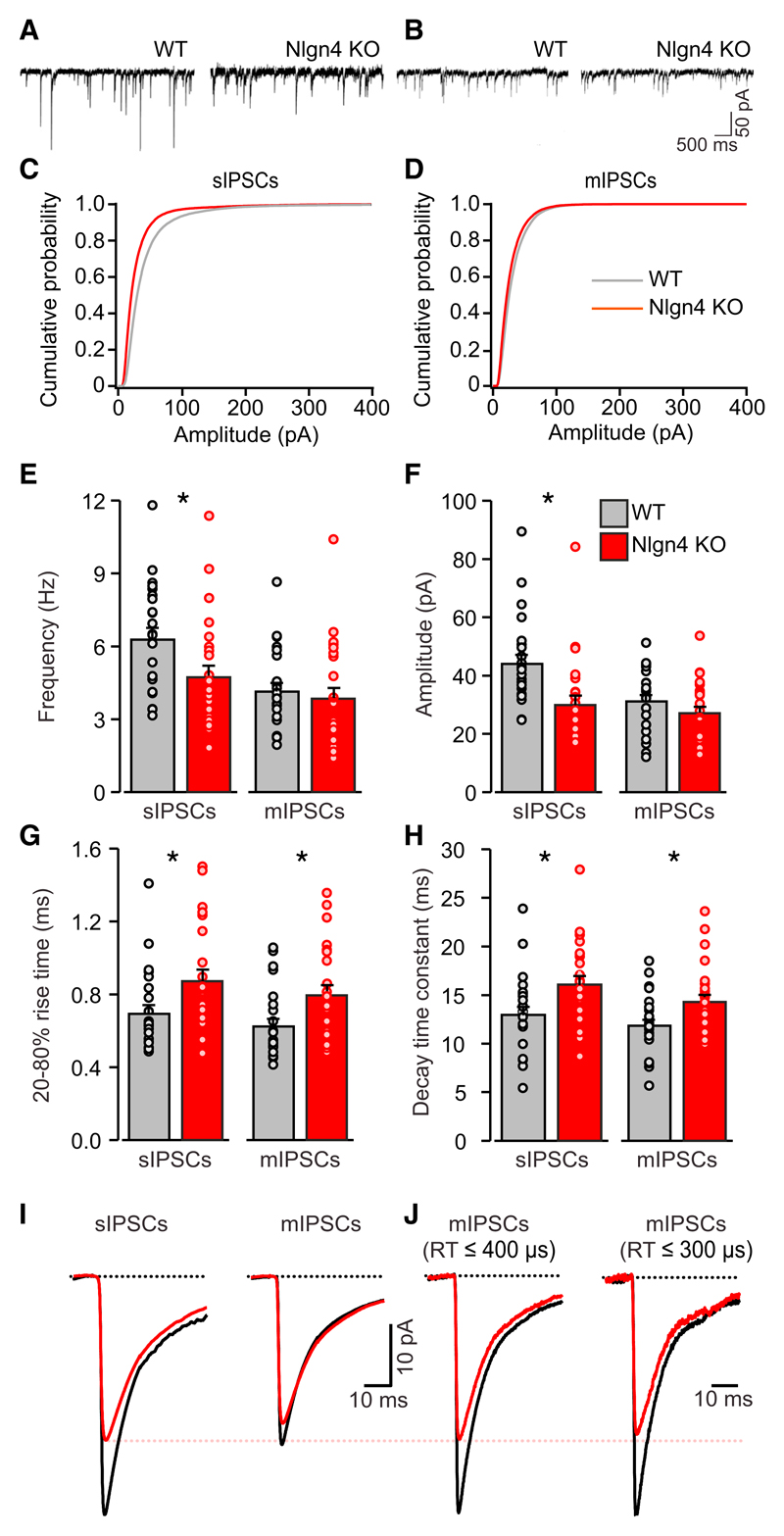
Inhibitory Synaptic Transmission in CA3 Pyramidal Cells of Nlgn4 KOs (A and B) Recordings of sIPSCs (A) and mIPSCs (B) from CA3 pyramidal neurons in acute hippocampal slices from WT and Nlgn4 KO mice are shown. (C and D) Comparisons of the average cumulative distribution of sIPSC amplitudes (C) and mIPSC amplitudes (D) are shown. (E–H) Quantifications of the average frequency (E), peak amplitude (F), 20%–80% RT (G), and decay time constant (H) of sIPSCs and mIPSCs are shown. (I) Normalized average sIPSCs and mIPSCs in WT (black) and Nlgn4 KO (red) mice are shown. (J) Normalized average current of mIPSCs having RTs ≤400 (left) and ≤300 μs (right) in WT (black) and Nlgn4 KO (red) mice is shown. Error bars represent SEM; n = 22 cells per genotype; *p < 0.05.

We next performed patch-clamp recordings from CA3 pyramidal cells in acute hippocampal slices of juvenile mice (post-natal day P12–P26) to test whether the altered composition of perisomatic inhibitory postsynapses in Nlgn4 KOs is accompanied by changes in synaptic transmission. We observed a significant decrease in the peak amplitude of spontaneous inhibitory post-synaptic currents (sIPSCs; Figures 3A, 3C, and 3F; WT, 45.2 ± 3.3 pA; KO, 31.0 ± 3.4 pA; n = 22, p < 0.01), as well as a slight but significant decrease in the sIPSC frequency (Figures 3A and 3C; WT, 6.4 ± 0.5 Hz; KO, 4.9 ± 0.5 Hz; n = 22, p < 0.05). No significant changes were observed in the amplitude or frequency of miniature IPSCs (mIPSCs; Figures 3B and 3D–3F; amplitude: WT, 32.2 ± 2.3 pA; KO, 28.2 ± 2.4 pA; n = 22; frequency: WT, 4.3 ± 0.4 Hz; KO, 4.0 ± 0.5 Hz; n = 22, not significant). The amplitude and frequency of spontaneous and miniature excitatory postsynaptic currents (sEPSCs and mEPSCs) remained unchanged (Figure S3), confirming that Nlgn4 KO affects primarily inhibitory synapses in area CA3 of the hippocampus.

Interestingly, analyses of sIPSC and mIPSC kinetics revealed a significant increase in the rise times (RTs) (Figure 3G) and decay time constants (Figure 3H) of sIPSCs (20%–80% RT: WT, 0.71 ± 0.05 ms; KO, 0.89 ± 0.06 ms; n = 22, p < 0.05; decay time constant: WT, 13.3 ± 0.9 ms; KO, 16.4 ± 0.9 ms; n = 22, p < 0.05) and mIPSCs (20%–80% RT: WT, 0.64 ± 0.05 ms; KO, 0.81 ± 0.06 ms; n = 22, p < 0.05; decay time constant: WT, 12.2 ± 0.7 ms; KO, 14.6 ± 0.8 ms; n = 22, p < 0.05). Among other possibilities, this slowing of the average time course of sIPSCs and mIPSCs could arise from a selective weakening of the inputs with the fastest kinetics, i.e., those that originate from perisomatic synapses (Kerti-Szigeti et al., 2014). We therefore re-analyzed two subsets of mIPSCs with RTs ≤400 and ≤ 300 μs, respectively (Figures 3I and 3J). This analysis revealed a decrease in the amplitude of the fastest mIPSCs (RT ≤ 400 μs: WT, 30.9 ± 3.1 pA; KO, 22.5 ± 3.1 pA; n = 22, p = 0.06; RT ≤ 300 μs: WT, 24.2 ± 2.6 pA; KO, 17.2 ± 2.0 pA; n = 22, p < 0.05) and a decrease in the fraction of fast events among the total number of events analyzed (RT ≤ 400 μs: WT, 18.1% ± 3.6%; KO, 8.2% ± 2.1%; n = 22, p < 0.05; RT ≤ 300 μs: WT, 6.3% ± 1.7%; KO, 2.6% ± 0.6%; n = 22, p = 0.05).

### Perturbed γ-Oscillations in the CA3 Region of Nlgn4 KO Hippocampus

**Figure 4:**
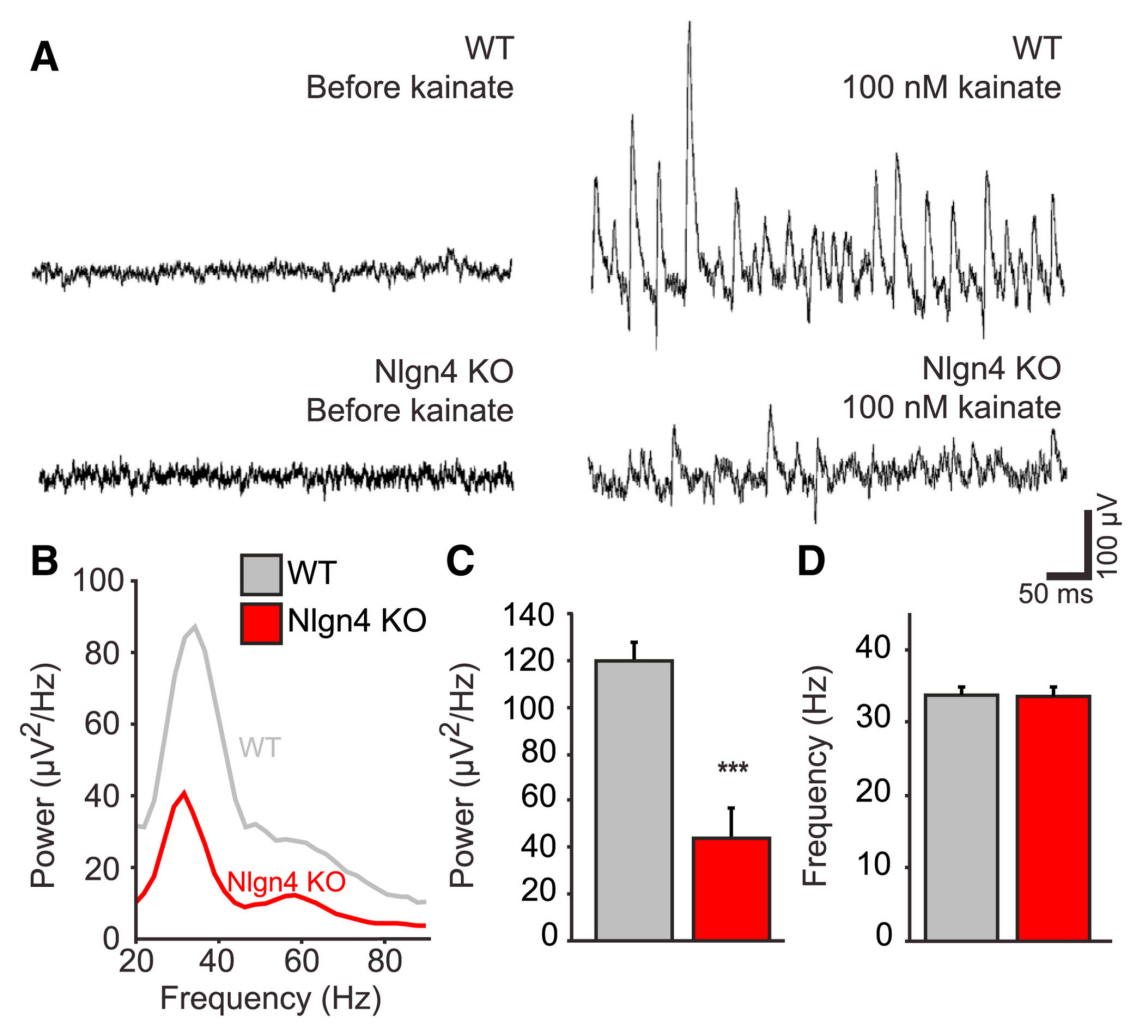
γ-Oscillations in the CA3 Region of Nlgn4 KOs (A) Traces of γ-oscillations induced by 100 nM kainate in acute CA3 hippocampal slices from WT and Nlgn4 KO mice are shown. (B) Representative power spectrum of γ-oscillations in WT (gray) and Nlgn4 KO (red) mice is shown. (C and D) Quantifications of the power (C) and frequency (D) of the maximum peak are shown. Error bars represent SEM; n = 6 mice per genotype (average of three slices/animal); ***p < 0.001.

Inhibitory connections in the hippocampus contribute substantially to the generation of synchronized network activity, particularly in the γ frequency range of 30–80 Hz (Bartos et al., 2007). These γ-oscillations, in turn, have been implicated in the cognitive functions mediated by the hippocampus (Uhlhaas and Singer, 2012). To investigate whether γ-oscillations are altered in Nlgn4 KOs, oscillatory activity was induced pharmacologically in acute hippocampal slices by kainate application (100 nM) (Fisahn et al., 2004). This treatment yielded long-lasting and stable oscillations within 10 min of drug application (Figure 4A). Power spectrum analyses revealed a substantial reduction of the power of oscillations in Nlgn4 KOs (Figures 4B and 4C; WT peak, 119.8 ± 8.4 μV^2^/Hz; KO peak, 43.9 ± 13.0 μV^2^/Hz; n = 6 pairs of WT and KO animals, three slices per animal, p < 0.001), while the frequency was not altered (Figure 4D; WT, 33.8 ± 1.1 Hz; KO, 33.6 ± 1.4 Hz; n = 6 pairs of WT and KO animals, three slices per animal). These findings are consistent with the notion that Nlgn4 loss may cause some of the cognitive impairments associated with ASD by perturbing oscillatory activity in the hippocampus and potentially in other brain regions, such as the prefrontal cortex.

## Discussion

This study shows that Nlgn4 loss subtly alters the composition of perisomatic inhibitory synapses and inhibitory synaptic transmission and strongly perturbs γ-oscillations in the mouse hippocampus CA3 region. The following three key conclusions arise from our findings: (1) Nlgn4, like Nlgn2, functions primarily at inhibitory synapses in this region, likely at perisomatic synapses originating from parvalbumin (PV)-positive interneurons; (2) subtle local changes in synapse function resulting from ASD-associated Nlgn4 mutations can accumulate to yield pronounced perturbations in global network activity; and (3) the resulting alterations in synchronized oscillatory activity may represent an important mechanism linking loss of Nlgn4 function to cognitive dysfunction in individuals with Nlgn4 mutations.

A first important result of our study is that Nlgn4 has a synapse type-specific function in the mouse hippocampus. We found that Nlgn4 KO causes a selective loss of components of inhibitory postsynapses in the stratum pyramidale of the hippocampal CA3 region (Figures 2 and S2); specific reductions in the amplitude and frequency of sIPSCs, but not sEPSCs, in CA3 pyramidal cells (Figures 3 and S3); and an upregulation of the Nlgn2/Nlgn1 ratio, indicating a compensation of Nlgn4 loss by the functionally related Nlgn2 (Table S1). These observations corroborate earlier cell biological studies, which showed that Nlgn4, like Nlgn2, can recruit GABA_A_Rs to membranes by CB activation and GPH binding; that Nlgn4 is localized to inhibitory postsynapses in retina, brainstem, and spinal cord; and that loss of Nlgn4 in retina causes a selective upregulation of Nlgn2 (Hoon et al., 2011). Interestingly, our findings are highly reminiscent of the effects of Nlgn2 KO (Jedlicka et al., 2011; Poulopoulos et al., 2009; Soykan et al., 2014), and together they indicate that Nlgn4, like Nlgn2, may function primarily at inhibitory synapses.

Whether Nlgn4 also plays a role at excitatory synapses is unclear, at least for the mouse brain. Excitatory synapses in mouse retina, brainstem, and spinal cord do not contain Nlgn4 (Hoon et al., 2011). Extrapolating from this observation, which is necessary due to detectability limitations, and considering that we found here that Nlgn4 loss affects only inhibitory, but not excitatory, transmission (Figures 2, 3, 4, and S3), it appears that Nlgn4 functions specifically at inhibitory synapses in the mouse hippocampus and that the slight decrease in the PSD-95 puncta density in the CA3 stratum lucidum of Nlgn4 KOs (Figure S2) may be a result of a compensatory homeostatic downregulation in response to the perturbation of inhibitory signaling. It is possible that a different scenario arises in mouse somatosensory cortex, where Nlgn4 KO was reported to affect both inhibitory and excitatory synapses (Delattre et al., 2013), but the present study did not provide supporting evidence.

The issue of synapse specificity of human NLGN4 is more complex. Of all Nlgns, Nlgn4/NLGN4 are least conserved between mouse and human (Bolliger et al., 2008; Jamain et al., 2008), and the subcellular localization of NLGN4 in the human brain is unknown. Expression of human NLGN4 in cultured mouse hippocampal neurons suppresses excitatory synaptic responses (Chanda et al., 2015), while NLGN4 expression in rat organotypic hippocampal slice cultures has the opposite effect (Bemben et al., 2015), indicating that NLGN4 can act at excitatory synapses if exogenously expressed. However, Nlgns have promiscuous synaptogenic effects when overexpressed in neurons (Chih et al., 2005), and Nlgn heterodimerization can further confound readouts of Nlgn specificity in overexpressing WT neurons (Poulopoulos et al., 2012). Final conclusions on the synapse specificity of human NLGN4 thus only are possible once its synaptic localization is known. Nevertheless, the conservation of GPH and, partially, of CB-binding sites between the mouse and human variants (Bolliger et al., 2008; Jamain et al., 2008), along with the fact that Nlgn4 KO mice show ASD-related phenotypes that are similar to the symptoms of NLGN4-deficient patients (Jamain et al., 2008), indicates that at least some key functions are conserved between mouse Nlgn4 and human NLGN4, including actions at inhibitory synapses in vivo.

Interestingly, our data indicate that the effects of Nlgn4 KO in hippocampal area CA3 are restricted to a highly specific subset of perisomatic inhibitory synapses in the stratum pyramidale. This layer specificity is reminiscent of the effects of Nlgn2 KO (Poulopoulos et al., 2009) and shows that these synapses depend on Nlgn4 while other synapses do not, likely because other adhesion systems govern their formation, maturation, and maintenance. This also is reflected by our electrophysiological analyses, which revealed decreased amplitudes and frequencies of sIPSCs along with increased sIPSC RTs and decay time constants in Nlgn4 KO pyramidal cells of the CA3 region, while mIPSCs had primarily slower kinetics and only in the fastest events also showed reduced amplitudes (Figure 3). These data indicate that Nlgn4 KO mainly affects synapses originating from interneuron subtypes that contribute strongly to spontaneous action-potential-driven inputs to somata of CA3 principal cells, have a low probability of spontaneous synaptic vesicle fusion (i.e., contribute few mIPSCs), and target principal cell somata or proximal dendrites—criteria that are met by PV-positive basket cells in several brain regions (Goswami et al., 2012; Katona et al., 2014; Lapray et al., 2012; Pfeffer et al., 2013; Wolff et al., 2014). Moreover, PV-positive basket cells contribute the majority of exactly those perisomatic inhibitory inputs of hippocampal pyramidal cells (Takács et al., 2015) that are specifically affected by Nlgn4 KO, and the kainate-induced γ-oscillations that are strongly affected in the Nlgn4 KOs (Figure 4) are highly dependent on the perisomatic synaptic inhibition generated by PV-positive interneurons (Bartos et al., 2007; Cardin et al., 2009; Mann and Paulsen, 2007; Sohal et al., 2009). Together, these considerations lead us to conclude that Nlgn4 KO mainly perturbs the inputs of PV-positive interneurons to CA3 principal cells.

Beyond this notable synapse specificity, the effects of Nlgn4 loss on inhibitory synapses in the hippocampal CA3 region, while clearly discernible, are relatively subtle even at the affected subset of synapses (Figures 2 and 3). This is perhaps unsurprising given that Nlgn4 is much less strongly expressed in mouse brain than other Nlgns (Varoqueaux et al., 2006). Strikingly, however, despite affecting only an estimated 1.5% of the total inhibitory synapse population in area CA3, these small changes in sum appear to cause large perturbations in the overall activity of the network, as indicated by the pronounced reduction in the power of the γ-oscillations in the Nlgn4 KO. The γ-oscillations are thought to play a key role in the cognitive functions mediated by the hippocampus (Uhlhaas and Singer, 2012), including sensory processing and memory formation, and they have been correlated with specific behavior conditions, such as exploratory behavior (Bragin et al., 1995) and performance during working memory tasks (Fuchs et al., 2007). Our findings thus provide an interesting potential mechanism by which seemingly minor molecular and functional synaptic abnormalities induced by disease-associated Nlgn4 mutations may have a substantial impact on functional psychiatric phenotypes. While further experiments will be necessary to confirm this hypothesis, such mechanisms may be of widespread relevance to ASD given the subtle nature of most neuropathological alterations identified in postmortem tissue from ASD patients to date (Chen et al., 2015).

Indeed, weaker γ-oscillations are observed in several neuropsychiatric disorders, including ASD (Maxwell et al., 2015). There are, thus, striking links between ASD and aberrant γ-oscillations in human patients on the one hand and between ASD-related human loss-of-function mutations in *NLGN4* and aberrant GABAergic synaptic transmission and γ-oscillations in a mouse model of ASD-related Nlgn4 loss on the other hand. These links raise the possibility that the behavioral abnormalities due to NLGN4/Nlgn4 loss in ASD patients and model mice are caused by aberrant GABAergic signaling and γ-oscillatory activity, and may thus be treatable by therapies that target the GABAergic system in the oscillatory circuitry and restore synchronized network activity in the hippocampus and other brain regions.

This scenario may even have more general relevance to ASD and the development of novel treatments. Indeed, deficient inhibitory GABAergic signaling and alterations in PV-expressing interneurons also have been observed in other mouse models of ASD (Chao et al., 2010; Gogolla et al., 2009; Rothwell et al., 2014; Tyzio et al., 2014) as well as in postmortem tissue from individuals with ASD (Chen et al., 2015; Voineagu et al., 2011), and GABAergic signaling is emerging as a key treatment target in ASD (Braat and Kooy, 2015; Cellot and Cherubini, 2014; Möhler, 2015).

## Experimental Procedures

### Animals

Experiments were performed on juvenile (P12–P26) or adult (8- to 12-week-old) male Nlgn4 KOs and male WT littermates on a C57BL/6J background (see the Supplemental Experimental Procedures). All animal experiments were approved by the responsible government institution (Niedersächsisches Landesamt für Verbraucherschutz und Lebensmittelsicherheit).

### X-Gal Staining, Immunohistochemistry, and Immunoblotting

X-gal staining was conducted to assess Nlgn4 expression based on a β-galactosidase coding sequence in the gene trap insertion used to generate the Nlgn4 KO (Jamain et al., 2008). Immunohistochemical analysis of postsynaptic proteins was performed on methanol-fixed fresh-frozen brain sections. Immunohistochemical analysis of presynaptic proteins was performed on perfusion-fixed free-floating brain sections. Images were obtained using a TCS-SP5 inverted confocal microscope (Leica Microsystems) and a 63× water immersion objective. For quantification and colocalization experiments, images were processed using ImageJ. Immunoblotting of tissue from hippocampal subregions and synaptosomes was conducted according to standard procedures (see the Supplemental Experimental Procedures).

### Electrophysiology

Transverse hippocampal slices (300 μm thick) were obtained from juvenile mice. The sIPSCs, mIPSCs, sEPSCs, and mEPSCs were recorded in CA3 pyramidal cells in the whole-cell configuration. The γ-oscillations were recorded in area CA3 as extracellular field potentials under interface conditions (see the Supplemental Experimental Procedures).

### Statistics

Data are presented as mean ± SEM. Statistical significance of differences was evaluated with the two-tailed unpaired Student’s t test and p < 0.05 was taken as the level of statistical significance.

## Supplementary Material

Supplemental Information includes Supplemental Experimental Procedures, three figures, and one table and can be found with this article online at http://dx.doi.org/10.1016/j.celrep.2015.09.011.

Supplementary information

## Highlights

- Nlgn4 KO alters the composition of perisomatic inhibitory synapses in hippocampus
- Inhibitory synaptic transmission is impaired in Nlgn4 KO hippocampal area CA3
- Nlgn4 KO strongly perturbs oscillatory network activity in hippocampal area CA3
- Altered GABAergic signaling may link Nlgn4 loss-of-function mutations to autism
