# Supplementary material for: Perturbed Hippocampal Synaptic Inhibition and γ-Oscillations in a Neuroligin-4 Knockout Mouse Model of Autism
Source: Cell Rep. Author manuscript; Available in PMC 2018 Mar 21. (PMC5862414; doi:10.1016/j.celrep.2015.09.011)
Supplement: Supplementary information [file NIHMS76716-supplement-Supplementary_information_.pdf]

SUPPLEMENTAL DATA

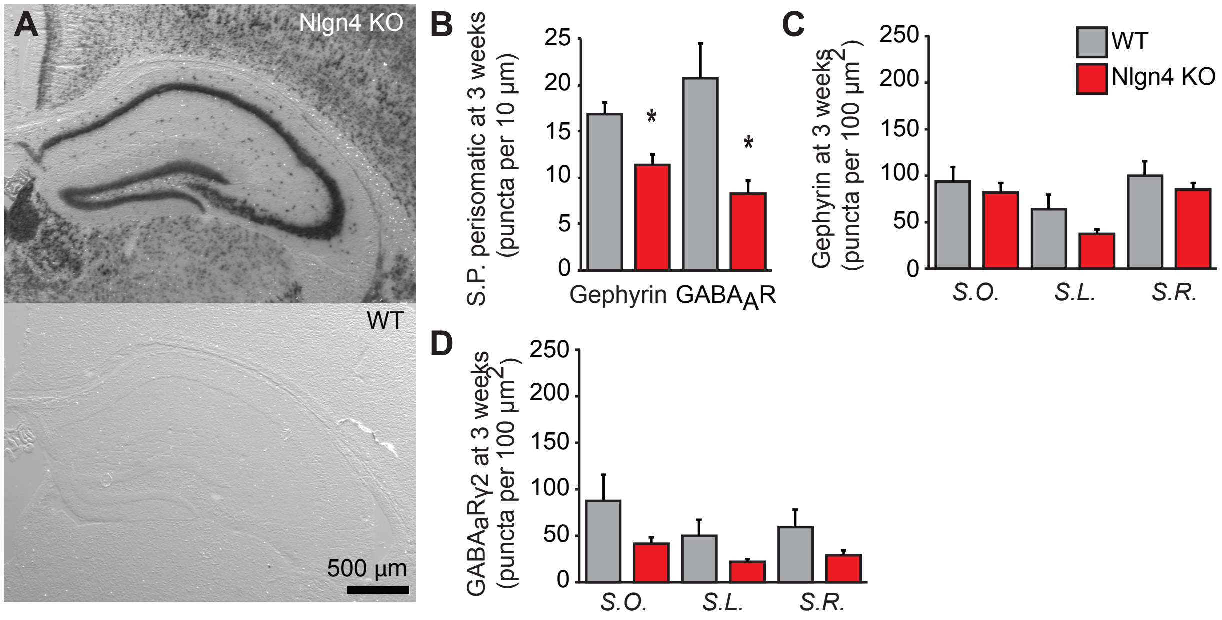

Figure S1 (Relates to Figures 1 and 2). Juvenile Mice Exhibit Similar Nlgn4 Expression Pattern and Inhibitory Postsynaptic Defects in the Hippocampus as Adult Mice.

(A) β-Galactosidase activity under the control of the Nlgn4 promoter reveals expression of Nlgn4 throughout the hippocampus. Scale bar, 500 µm. (B) Quantification of Gephyrin and $GABA_AR$ γ2 puncta in the perisomatic area of pyramidal cells in CA3 stratum pyramidale (puncta per 10 µm cell perimeter).

(C, D) Quantification of Gephyrin (C) and $GABA_AR$ γ2 subunit (D) puncta density in the stratum oriens, stratum lucidum, and stratum radiatum of WT and Nlgn4 KO CA3 (puncta per 100 μm^2).

n=9 pairs of mice at 3 weeks of age; error bars represent SEM; *, $p<0.05$.

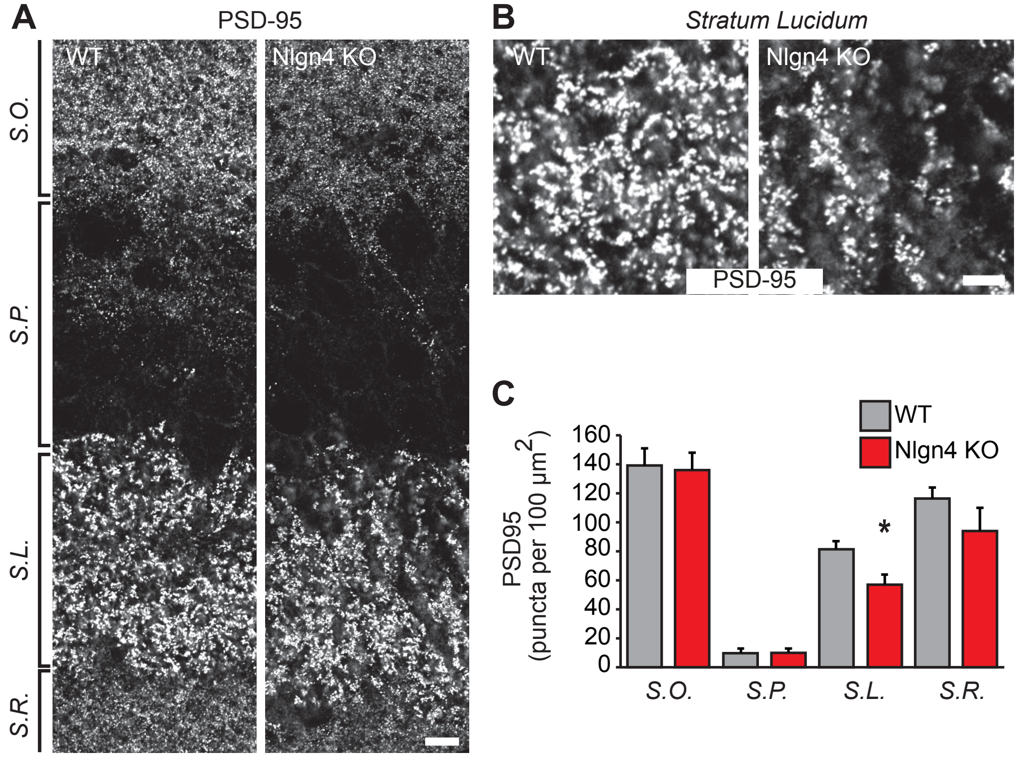

Figure S2 (Relates to Figure 2). Quantification PSD-95 Puncta in Hippocampal Area CA3 of WT and Nlgn4 KO Mice.

(A, B) Representative images showing PSD-95 immunoreactivity in CA3 sections (A) and in stratum lucidum (B) of adult WT and Nlgn4 KO mice. Scale bars, 10 µm (A) and 5 µm (B).

(C) Quantification of PSD-95 puncta density in the stratum oriens, stratum pyramidale, stratum lucidum, and stratum radiatum of WT and Nlgn4 KO mice (puncta per 100 μm^2).

n=9 pairs of mice; error bars represent SEM; *, $p<0.05$.

S.O., stratum oriens; S.P., stratum pyramidale; S.L. stratum lucidum; S.R., stratum radiatum.

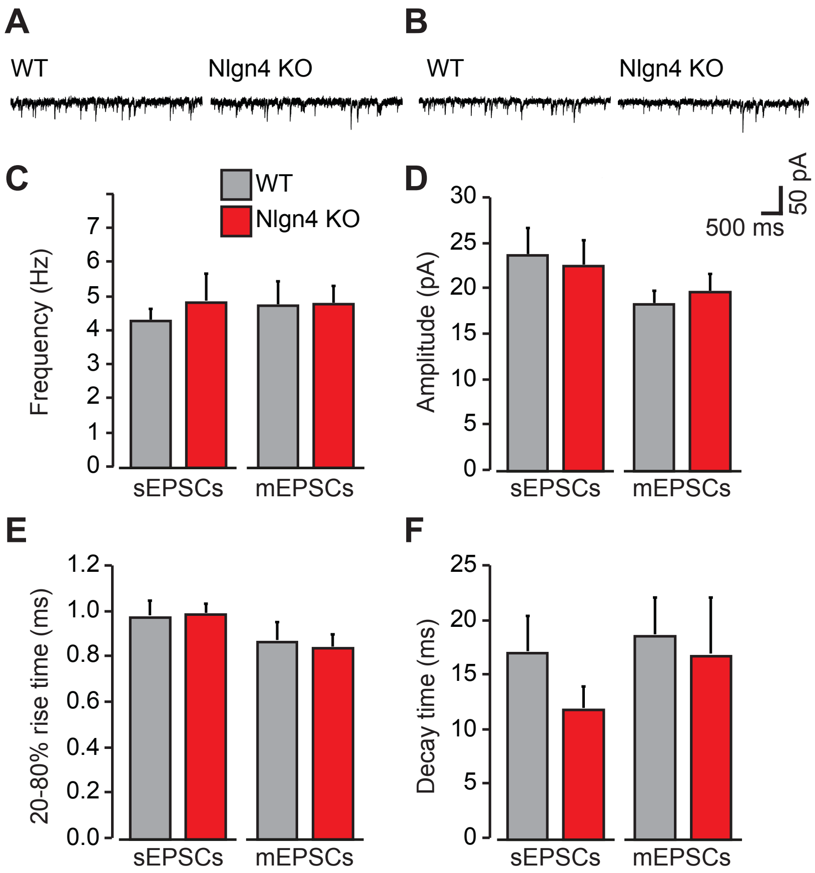

Figure S3 (Relates to Figure 3). Nlgn4 KO Mice Show Unaltered Excitatory Synaptic Transmission.

(A, B) Representative recordings of sEPSCs (A) and mEPSCs (B) from CA3 pyramidal neurons in acute hippocampal slices from WT and Nlgn4 KO mice.

(C, D) Quantification of the average frequency (C) and peak amplitude (D) of sEPSCs and mEPSCs in WT and Nlgn4 KO mice.

(E, F) 20-80% rise time (E) and decay time constant (F) of sEPSCs and mEPSCs in WT and Nlgn4 KO mice.

n=13 cells per genotype; error bars represent SEM; *, $p<0.05$.

Table S1 (Relates to Figure 2). Quantification of Protein Levels in Hippocampal Synaptosomes from WT and Nlgn4 KO Mice.

Synaptosomes	WT			KO				
	Mean	±	SEM	Mean	±	SEM	p value	n
Excitatory synapses								
PSD-95	100	±	4.4	99.3	±	3.6	0.848	18
GluN1	100	±	2.5	100.8	±	3.3	0.809	17
GluA1	100	±	4.3	103.2	±	3.9	0.478	16
GluA2	100	±	4.4	101.1	±	4.0	0.797	12
VGlut1	100	±	6.6	100.6	±	5.6	0.926	16
Inhibitory synapses								
Gephyrin	100	±	2.5	98.5	±	2.9	0.714	14
$GABA_AR$ α1	100	±	6.6	105.0	±	7.8	0.597	14
VIAAT	100	±	4.8	99.3	±	3.1	0.862	17
Neuroligins								
Nlgn1	100	±	3.5	92.9	±	4.1	0.081	16
Nlgn2	100	±	3.1	108.5	±	4.0	0.112	16
Ratio Nlgn2/Nlgn1	100	±	2.4	110,8	±	3,6	<0.05	16

Synaptosomes were prepared from whole hippocampus of adult mice and were assessed for levels of synaptic proteins by immunoblotting. Data are expressed as percentage of the WT average (i.e. individual sample value*100/average WT value) to facilitate comparison between WT and KO while maintaining the variance of the WT group.

SUPPLEMENTAL EXPERIMENTAL PROCEDURES

Animals

Nlgn4 KO mice (Jamain et al., 2008) and WT littermates were bred on a C57BL/6J background from heterozygous breeding pairs. Experiments were performed on juvenile (postnatal day P12-26) or adult (8-12 week old) male mice as specified below. Experimenters were blind to genotype at all stages of the experiment. All experiments were carried out in accordance with the German laws governing the use of laboratory animals.

X-Gal Staining

Expression of Nlgn4 in brain was assessed by taking advantage of the presence of a β-galactosidase (β-gal) coding sequence in the gene trap insertion used to generate the Nlgn4 KO (Jamain et al., 2008). Due to this insertion, Nlgn4 KOs express the β-gal under the control of the endogenous Nlgn4 promoter, which can be detected using 5-bromo-4-chloro-3-indolyl-β-D-galactopyranoside (X-gal) staining. Adult or juvenile animals (Nlgn4 KO mice expressing β-gal and WT controls not expressing β-gal) were deeply anesthetized with isoflurane (DeltaSelect) and decapitated. The brain was removed from the skull and rapidly frozen in a -35°C isopentane bath (Carl Roth). Brains were subsequently mounted on a specimen disc and embedded in Tissue-Tek®, and coronal brain sections (16 µm) were cut using a cryostat (Leica CM3050 S). Sections were directly mounted on glass slides and dried for 30 min at room temperature. Slides were submerged in 4% paraformaldehyde in 0.1 M PB for 10 min and washed with PBS for 3 x 5 min. Slides were incubated in a 2.5 mM X-gal solution overnight at 37°C. The slides were washed with PBS for 3 x 5 min before mounting using Aqua Poly/Mount (Polysciences). Low-magnification images were captured with a binocular microscope (Leica MZ16).

Immunohistochemistry

Immunohistochemistry for postsynaptic proteins was performed on methanol-fixed fresh frozen brain sections using a modification of a published protocol (Schneider Gasser et al., 2006). Adult or juvenile animals were deeply anesthetized with isoflurane (DeltaSelect) and decapitated. The brain was removed from the skull and rapidly frozen in a -35°C isopentane bath (Carl-Roth). Brains were subsequently mounted on a specimen disc and embedded in Tissue-Tek®, and coronal brain sections (16 µm) were cut using a cryostat (Leica CM3050 S). Sections were directly mounted on glass slides and dried for 30 min at room temperature. Slides were submerged in a -20°C methanol bath for 5 min, then washed for 3 x 5 min with PBS, and blocked for 1 h with blocking solution (3% goat serum and 0.2% Triton X-100 diluted in PBS). Slides were subsequently incubated overnight at 4°C with one of the following primary antibodies diluted in blocking solution: Anti-postsynaptic density 95 kDa antibody (PSD-95; mouse monoclonal, Neuromab, clone K28/43, 1/3000), anti-Gephyrin antibody (mouse monoclonal, Synaptic Systems, clone 3b11, 1/1000), and anti-$GABA_AR\gamma_2$ antibody ($GABA_AR\gamma_2$, polyclonal guinea pig, kindly provided by Prof. Dr. J.-M. Fritschy, 1/1000). After three washes with PBS, the tissue was incubated with secondary antibodies (goat anti-mouse or goat anti-guinea pig labeled with Alexa Fluor Dyes A488 or A555, Invitrogen, 1/1200) for 2 h at room temperature. Sections were then washed 3 x 5 min with PBS and sealed with mounting medium Aqua Poly/Mount (Polysciences) for imaging. Immunohistochemistry for presynaptic proteins was performed on perfusion-fixed free-floating brain sections. Adult or juvenile animals were anaesthetized using Avertin (2,2,2-tribromoethyl alcohol, final concentration 2% in tert-amyl alcohol/PBS, 10 ml/kg i.p.) and perfused transcardially with 4% paraformaldehyde in 0.1 M PB for 8 min. Brains were extracted and post-fixed in the same solution for 2 h. The perfused brains were then cryoprotected by successive incubation in sucrose solutions of increasing concentration (10, 20, and 30% in PBS). For sectioning, brains were frozen in an isopentane bath and mounted as described above, and coronal brain sections

(35 μm) were cut using a cryostat (Leica CM3050 S). Free-floating sections were washed for 3 x 5 min with PBS, blocked for 1 h in blocking solution as above, and incubated overnight at 4°C with anti-vesicular inhibitory amino acid transporter (VIAAT) antibody (rabbit polyclonal, Synaptic Systems, 1/1000). After three consecutive washes with PBS, sections were incubated with secondary antibodies (goat anti-guinea pig labeled with Alexa Fluor Dye A488, Invitrogen, 1/1200) for 2 h at room temperature. Sections were then washed 3 x 5 min in PBS, mounted on slides, allowed to dry for 15 min, and sealed with mounting medium Aqua Poly/Mount (Polysciences) for imaging and quantification.

Image Acquisition

For synaptic quantification and localization, high magnification single-plane confocal images of hippocampal area CA3a/b were obtained using a TCS-SP5 inverted confocal microscope (Leica Microsystems) and a 63X water immersion objective. A digital zoom factor of 1 was used to obtain low magnification images for protein localization within a brain region, while a zoom factor of 8 was used for the high magnification images necessary for synaptic quantification. The gain and offset settings during image acquisition were defined for each antibody and magnification in order to obtain equivalent pictures for all animals.

Image Analysis and Presentation

All figures were prepared using Photoshop CS5 (Adobe). The only modifications from the original images were readjustments of the brightness, contrast, and tonal range, and identical settings were always applied to matched pairs of WT and Nlgn4 KO mice. For quantification and colocalization experiments, images were processed using Image J (Schneider et al., 2012). Each image was first thresholded to generate a binary image, in which individual synapses were represented by single

particles, The same threshold was applied to all images and genotypes within the same brain region. The binarized images were then subjected to the Watershed Segmentation algorithm to automatically separate overlapping particles. Finally, the number of synapses per image was counted using Image Js 'Analyze Particles' function. A synapse was defined as a particle consisting of 10 or more adjoining pixels (2 μm). To quantify perisomatic synapses in the stratum pyramidale, the perisomatic area was defined by manually tracing the perimeter of the cell. An area of interest was then defined that included a 1.75 μm wide zone on either side of the perimeter, i.e. 3.5 μm in total width. The puncta present in this area of interest were quantified and defined as synapses (6 cells per animal).

Preparation of Hippocampal Tissue for Immunoblotting

To quantify levels of Nlgn4 expression in WT hippocampus, adult mice were sacrificed by rapid decapitation, and the brains were removed from the skull and placed in high sucrose dissection buffer on ice (87 mM NaCl, 2.5 mM KCl, 1.25 mM KH_2PO_4, 25 mM $NaCO_3$, 0.5 mM $CaCl_2$, 7 mM $MgCl_2$, 75 mM Sucrose, 10 mM Glucose, 1.3 mM ascorbic acid, supplemented with protease inhibitors, Calbiochem). The brains were transferred into a mouse brain matrix (Braintree Scientific), and 1 mm coronal sections were prepared and immediately frozen on dry ice. The brain sections were then partially thawed and specific hippocampal regions of interest were rapidly isolated (CA1, CA3, dentate gyrus). These regions were rapidly frozen in liquid nitrogen and stored at -80°C until they were homogenized in RIPA buffer (50 mM Tris, 150 mM NaCl, 0.1% SDS, 0.5% sodium deoxycholate, 1% Triton X-100, supplemented with protease inhibitors, Calbiochem) using an all-glass dounce homogenizer (0.013-0.064 mm clearance, Kontes Glass Co.) and processed for immunoblotting. Synaptosomal fractions were prepared according to a modification of a published protocol (Carlin et al., 1980). Mice were sacrificed by rapid decapitation, brains were removed, and hippocampi were dissected out on ice. All further steps were performed at 4°C. Hippocampi were homogenized in

solution A (0.32 M sucrose, 1 mM $MgCl_2$, 0.5 mM $CaCl_2$, 1 mM HEPES, pH 7.4, containing freshly added protease inhibitor cocktail, EMD Biosciences) using a motor-operated Teflon/glass homogenizer (Potter S, Sartorius) with 12 strokes at 900 rpm. Homogenates were centrifuged for 10 min at 3000 rpm (approx. 1000 x g), and supernatants were saved. Pellets were resuspended in solution A, re-homogenized with 3 strokes at 900 rpm, and once again centrifuged for 10 min at 3000 rpm. The supernatants from the two centrifugation steps were pooled and centrifuged for 10 min at 10000 rpm (approx. 10000 x g). Pellets were resuspended in solution B (0.32 M sucrose, 1 mM HEPES, pH 7.4 containing freshly added protease inhibitor cocktail, EMD Biosciences) with 4-5 strokes at 900 rpm and layered over a sucrose gradient consisting of 0.85 M/1.0 M/1.2 M sucrose layers (each containing 1 mM HEPES and protease inhibitor cocktail, EMD Biosciences). Gradients were centrifuged for 2 h at 21600 rpm (approx. 82500 x g) using an SW 40 Ti rotor (Beckman Coulter). The band at the interface between 1.0 M and 1.2 M sucrose containing the synaptosomes was collected. SDS was added to a final concentration of 1%, and lysed synaptosomes were stored at -80°C until they were processed for immunoblotting.

Immunoblot Analysis

Total protein concentration of each sample was determined using a BCA assay (Pierce). A protein concentration corresponding to approximately 10 μg per sample was loaded for SDS-PAGE (Laemmli, 1970). Proteins were denatured by heating to 95°C in Laemmli buffer (2% SDS, 62.5 mM Tris, 10% glycerol, 1% β-mercaptoethanol, 0.01% bromophenol blue, pH 6.8) for 5 min. Samples were resolved on 10% SDS-PAGE gels, transferred onto nitrocellulose membranes by wet transfer (Towbin et al., 1979) and stained for total protein using a Memcode assay (Pierce). After evaluation of the total protein concentration per sample, nitrocellulose membranes were blocked in 50% LiCor blocking buffer (LiCor Biosciences) in PBS for 1 h and incubated with primary antibodies in primary antibody dilution

buffer (50% LiCor blocking buffer and 0.1% Tween-20 in PBS) under agitation at 4°C overnight. The following very well validated primary antibodies were used: anti-PSD-95 (mouse monoclonal, Neuromab, clone K28/43, 1/50000), anti-GluN1 (mouse monoclonal, Synaptic Systems, 1/1000), anti-GluA1 (rabbit polyclonal, Chemicon, 1/1000), anti-GluA2 (rabbit polyclonal, Synaptic Systems, 1/1000), anti-VGluT1 (guinea pig polyclonal, Synaptic Systems, 1/1000), anti-Gephyrin (mouse monoclonal, Synaptic Systems, clone 3B11, 1/1000), anti-$GABA_AR$ $\alpha2$ (rabbit polyclonal, Millipore, 1/1000), anti-VIAAT (guinea pig polyclonal, Synaptic Systems, 1/1000), anti-Nlgn1 [mouse monoclonal, clone 4C12, 1/1000, (Song et al., 1999)], anti-Nlgn2 [rabbit polyclonal #799, 1/1000, (Varoqueaux et al., 2004)], anti-Nlgn4 [rabbit polyclonal #76, 1/500, (Hoon et al., 2011; Jamain et al., 2008)]. After washing for 3 x 5 min with 0.1% Tween-20 in PBS, the membranes were incubated with secondary antibody in secondary antibody dilution buffer (50% LiCor blocking buffer, 0.1% Tween-20 and 0.01% SDS in PBS) under agitation for 1 h at room temperature. The following secondary antibodies were used: Goat anti-mouse Alexa680 (Invitrogen, 1/2500), goat anti-rabbit IRDye800 (Rockland Immunochemicals, 1/2500), goat anti-guinea pig IRDye700 (Rockland Immunochemicals, 1/2500). Blots were then washed as previously and scanned on an Odyssey Infrared Imager (LiCor Biosciences). Signal intensity for each sample was quantified using the Odyssey 2.0 software. Each sample intensity value was divided by the total protein loading value for the corresponding lane and then normalized to the average sample value of all lanes on the same blot to correct for blot-to-blot variance as described previously (Krueger et al., 2011). Data are expressed as percentage of the WT average (i.e. individual sample value*100/average WT value) to facilitate comparison between WT and KO while maintaining the variance of the WT group.

Electrophysiology

Juvenile (P15-26) mice were anesthetized with isoflurane (DeltaSelect) and decapitated. Brains were

removed from the skull and placed into a sucrose-based slicing solution on ice (230 mM sucrose, 26 mM $NaHCO_3$, 2 mM KCl, 1 mM KH_2PO_4, 2 mM $MgCl_2$, 10 mM D-glucose, 0.5 mM $CaCl_2$) and oxygenated with carbogen gas (95% O_2, 5% CO_2). Transverse hippocampal slices (300 μm thick) were obtained as published (Bischofberger et al., 2006) using a vibratome (Leica VT1200S). The sections were transferred to a holding chamber and maintained in ACSF (120 mM NaCl, 26 mM $NaHCO_3$, 1 mM KH_2PO_4, 2 mM KCl, 1 mM $MgCl_2$, 10 mM D-glucose, 2 mM $CaCl_2$). Before recording, sections were allowed to recover for ≥15 min. All recordings were performed in hippocampal area CA3a/b, where the peak power of kainate-induced γ-oscillations is largest (Craig and McBain, 2015).

Whole-cell patch-clamp recordings were obtained at room temperature (22°C) using a EPC10 amplifier (HEKA). Data acquisition was performed using Patchmaster software (HEKA). Neurons chosen for recording had morphological properties of mature pyramidal cells. The pipette solution for recording postsynaptic currents contained 140 mM KCl, 10 mM EGTA, 2 mM $MgCl_2$, 2 mM Na_2ATP, and 10 HEPES, pH adjusted to 7.2 with KOH, osmolarity adjusted to 315 mOsm/L with sucrose. Patch pipettes were pulled from thick-walled borosilicate glass tubing. When filled with internal solution, their resistance was 2-4 MΩ. Series resistance (Rs) was 6–12 MΩ. R_s was not compensated, but carefully monitored during the experiments. The holding potential was set to -70 mV in all experiments. Cells with a holding current larger than -120 pA were discarded. Spontaneous IPSCs (sIPSCs) were recorded from CA3 pyramidal cells in the presence of 10 μM 6-cyano-7-nitroquinoxaline-2,3-dione (CNQX), and 20 μM D-2-amino-5-phosphonopentanoic acid (D-AP5) to block excitatory postsynaptic currents. Spontaneous EPSCs (sEPSCs) were recorded in the presence of 10 μM bicuculline methiodide to block inhibitory postsynaptic currents. Tetrodotoxin (TTX) at 1 μM was added to the bath solution when recording miniature IPSCs (mIPSCs) or miniature EPSCs (mEPSCs). Spontaneous and miniature synaptic currents were captured using a template matching algorithm (Pernía-Andrade et al., 2012) implemented in Axograph X software (Axograph Scientific).

Further analysis was done using Axograph X or IgorPro (Wavemetrics) software. Decay time constants of sIPSCs and mIPSCs were obtained for each cell from single-exponential fits to the decay of the average IPSC waveforms.

For recording γ-oscillations in hippocampal slices, an interface-type recording chamber (BSC-BU Base Unit with the BSC-HT Haas Top, Harvard Apparatus) was used. Slices were placed on a nylon mesh at the interface of a stream of extracellular recording solution and a stream of warm, moist carbogen gas. The temperature of the stream was controlled to be at 33°C. Extracellular recording electrodes with a resistance between 1.5–3.5 MΩ were filled with extracellular recording solution and placed in the hippocampal pyramidal cell layer of CA3. Extracellular field potentials were recorded with a 700B amplifier (Axon Instruments, Molecular Devices) and the Digidata 1440A data acquisition system (Axon Instruments, Molecular Devices), and low-pass filtered at cut-off frequency of 3 kHz using a Bessel filter. To induce γ-oscillations, 100 nM kainate were applied. Oscillatory activity could generally be recorded ≥10 min after the start of kainate application. For each slice, baseline field potentials were recorded for 30 min, followed by 30 min recording of γ-oscillation field potentials during application of 100 nM kainate. Data were analyzed using Axograph X software (AxoGraph Scientific). Power spectra were calculated for 10-min epochs (last 10 min of each recording) of recorded field activity. The baseline power spectrum was subtracted from the power spectrum obtained during kainate application. The power and frequency within the band range of 25-45 Hz was calculated as the area under the respective peak in the power spectrum.

Statistics

All data are presented as mean ± SEM. Statistical significance of difference was evaluated with the two-tailed unpaired Student's t-test (immunohistochemistry and electrophysiology data), two-tailed paired Student’s t-test (synaptosome data except for Nlgn2/Nlgn1 ratio) or Mann Whitney U test

(synaptosome Nlgn2/Nlgn1 ratio to account for non-normal distribution of data). $p < 0.05$ was taken as the level of statistical significance.

SUPPLEMENTAL REFERENCES

Bischofberger, J., Engel, D., Li, L., Geiger, J.R.P., and Jonas, P. (2006). Patch-clamp recording from mossy fiber terminals in hippocampal slices. Nat. Protoc. *1*, 2075-2081.

Carlin, R.K., Grab, D.J., Cohen, R.S., and Siekevitz, P. (1980). Isolation and characterization of postsynaptic densities from various brain regions: enrichment of different types of postsynaptic densities. J. Cell. Biol. *86*, 831-845.

Craig, M.T., and McBain, C.J. (2015). Fast gamma oscillations are generated intrinsically in CA1 without the involvement of fast-spiking basket cells. J. Neurosci. *35*, 3616-3624.

Hoon, M., Soykan, T., Falkenburger, B., Hammer, M., Patrizi, A., Schmidt, K.F., Sassoe-Pognetto, M., Lowel, S., Moser, T., Taschenberger, H., Brose N., and Varoqueaux, F. (2011). Neuroligin-4 is localized to glycinergic postsynapses and regulates inhibition in the retina. Proc. Natl. Acad. Sci. USA *108*, 3053-3058.

Jamain, S., Radyushkin, K., Hammerschmidt, K., Granon, S., Boretius, S., Varoqueaux, F., Ramanantsoa, N., Gallego, J., Ronnenberg, A., Winter, D., Frahm, J., Fischer, J., Bourgeron, T., Ehrenreich, H., and Brose N. (2008). Reduced social interaction and ultrasonic communication in a mouse model of monogenic heritable autism. Proc. Natl. Acad. Sci. USA *105*, 1710-1715.

Krueger, D.D., Osterweil, E.K., Chen, S.P., Tye, L.D., and Bear, M.F. (2011). Cognitive dysfunction and prefrontal synaptic abnormalities in a mouse model of fragile X syndrome. Proc. Natl. Acad. Sci. USA *108*, 2587-2592.

Laemmli, U.K. (1970). Cleavage of structural proteins during the assembly of the head of bacteriophage T4. Nature *227*, 680-685.

Pernía-Andrade, A.J., Goswami, S.P., Stickler, Y., Fröbe, U., Schlögl, A., and Jonas, P. (2012). A deconvolution-based method with high sensitivity and temporal resolution for detection of spontaneous synaptic currents in vitro and in vivo. Biophys. J. *103*, 1429-1439.

Schneider, C.A., Rasband, W.S., and Eliceiri, K.W. (2012). NIH Image to ImageJ: 25 years of image analysis. Nat. Meth. *9*, 671-675.

Schneider Gasser, E.M., Straub, C.J., Panzanelli, P., Weinmann, O., Sassoe-Pognetto, M., and Fritschy, J.M. (2006). Immunofluorescence in brain sections: simultaneous detection of presynaptic and postsynaptic proteins in identified neurons. Nat. Protoc. *1*, 1887-1897.

Song, J.-Y., Ichtchenko, K., Südhof, T.C., and Brose, N. (1999). Neuroligin 1 is a postsynaptic cell-adhesion molecule of excitatory synapses. Proc. Natl. Acad. Sci. USA *96*, 1100-1105.

Towbin, H., Staehelin, T., and Gordon, J. (1979). Electrophoretic transfer of proteins from polyacrylamide gels to nitrocellulose sheets: procedure and some applications. Proc. Natl. Acad. Sci. USA *76*, 4350-4354.

Varoqueaux, F., Jamain, S., and Brose, N. (2004). Neuroligin 2 is exclusively localized to inhibitory synapses. Eur. J. Cell Biol. *83*, 449-456.
